# Supplementary material for: Potential Combination Drug Therapy to Prevent Redox Stress and Mitophagy Dysregulation in Retinal Müller Cells under High Glucose Conditions: Implications for Diabetic Retinopathy
Source: Diseases. 2021 Dec 14;9(4):91. doi: 10.3390/diseases9040091 (PMC8700204; doi:10.3390/diseases9040091)
Supplement: Supplementary file 1 [file diseases-09-00091-s001.zip › diseases-1406177-supplementary.pdf]

Supplementary Figures:

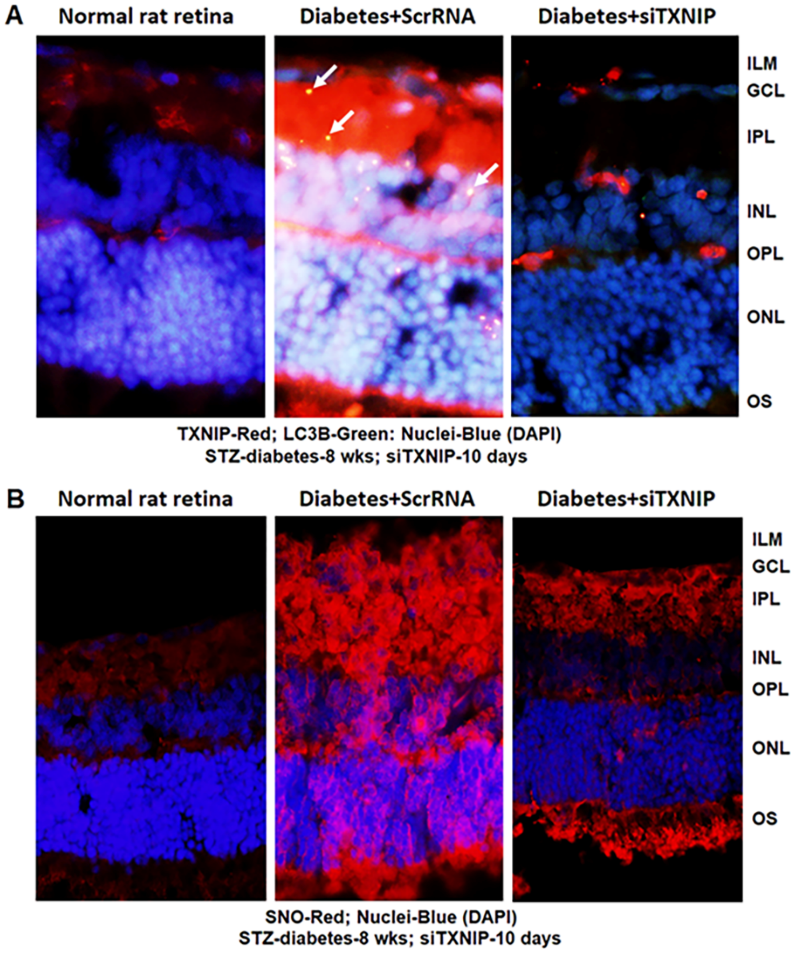

Figure S1. TXNIP knockdown prevents LC3B punctate and redox stress in the diabetic rat retina**.** (A) At 8 weeks of diabetes duration, TXNIP (red) is strongly induced in the diabetic rat retina when compared to the non-diabetic rat retina. In addition, LC3B punctate (yellow = LC3B green + TXNIP red) is seen in the diabetic retina. Both TXNIP and LC3B punctate are reduced by intravitreal siTXNIP injection, 10 days before terminating the experiment. (B) Similary, protein-s-nitrosylation (SNO) in diabetic retina is prevented by siTXNIP. Nuclei are counter-stained with DAPI (Blue). The images represent n = 3 of immunofluorescence staining, taken by an Olympus BX51 fluorescence microscope at 400× magnification.

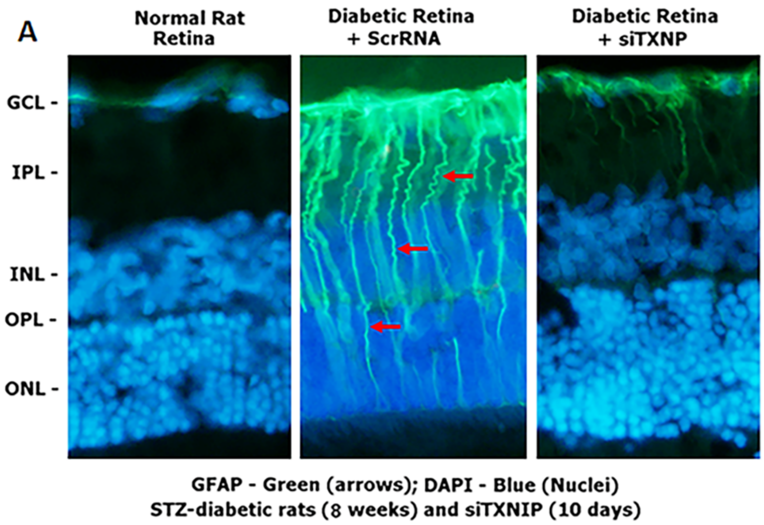

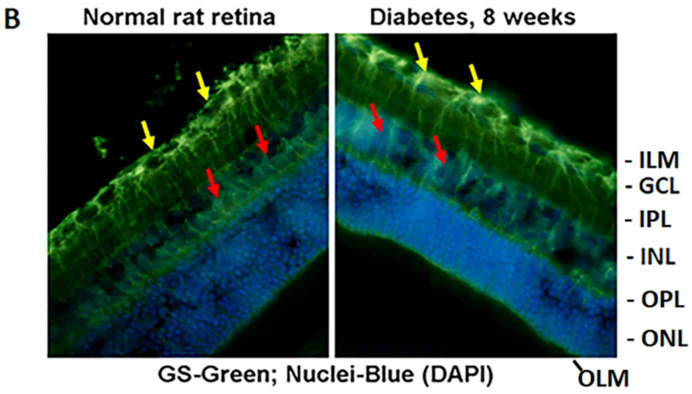

Figure S2. TXNIP knockdown prevents Müller glial activation in the diabetic rat retina. (A) At 8 weeks of diabetes duration, radial GFAP (green) is strongly induced in the neuroretina of the diabetic rats when compared to non-diabetic normal rat retina, suggesting retinal injury. Intravitreal TXNIP siRNA injection reduces GFAP expression. (B) Interestingly, the expression pattern of glutathione synthetase (GS) in the diabetic retina is seen to be redistributed towards the GCL (yellow arrows) from the soma at the IPL layer (red arrows), suggesting ganglion cell or axonal injury. Nuclei are counter-stained with DAPI (Blue). The images represent n = 3 of immunofluorescence microscopy, 400× magnification, taken by an Olympus BX51 fluorescence microscope.

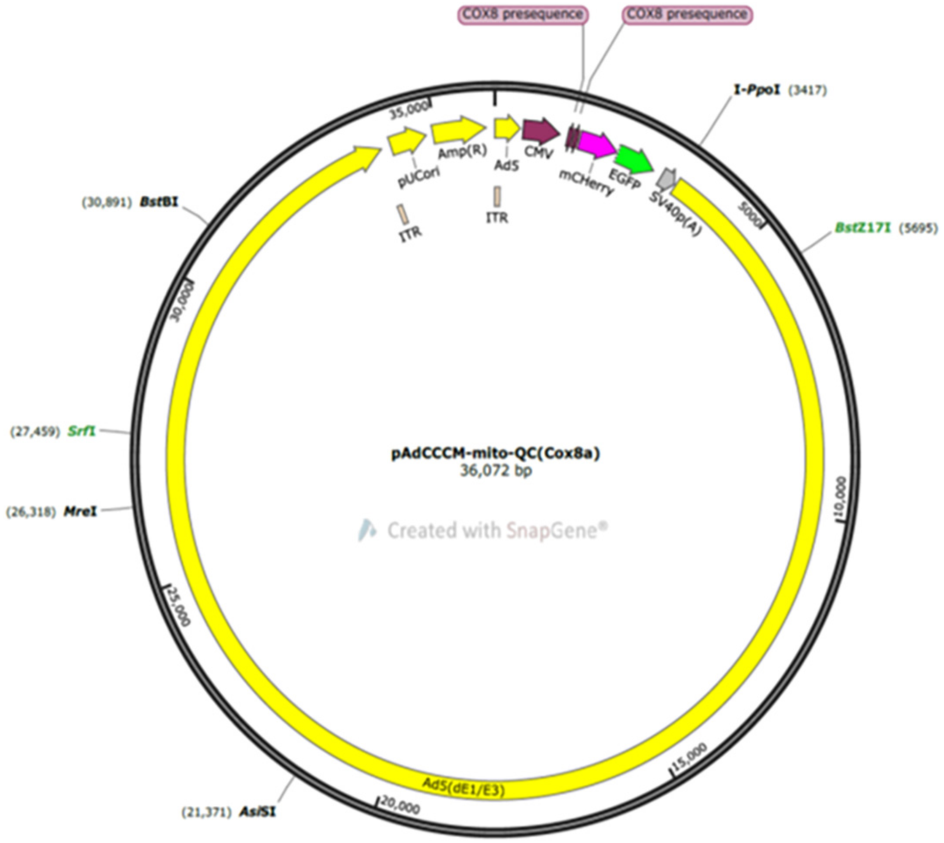

2xmt8a-CG

Figure S3. Diagram of the adenovirus-CMV-2xmt8a-mCherry-EGFP mitophagic probe (vector builders). This construct uses mitochondrial target or leader sequence of COX8a in tandem followed by mCherry and EGFP under the CMV promoter in an adenoviral vector. We named this probe as 2xmt8a-CG in order to distinguish from existing mito-QC that targets to the outer mitochondrial membrane using Fis1. When transducing this probe in cells, the mitochondrion appears yellow due to the expression of both mCherry (red) and EGFP (green) in live cells. On the other hand, after mitophagic flux to acidic lysosomes, EGFP fluorescence is quenched, therefore, emits red (mCherry alone), indicating a red mitolysosome. As EGFP fluorescence is not re-established once destroyed, the 2xmt8a-CG probe can also be used in fixed-cell imaging, an added advantage, if one wants to use in animal studies, where imaging multiple live tissues may be inconvenient or not possible.

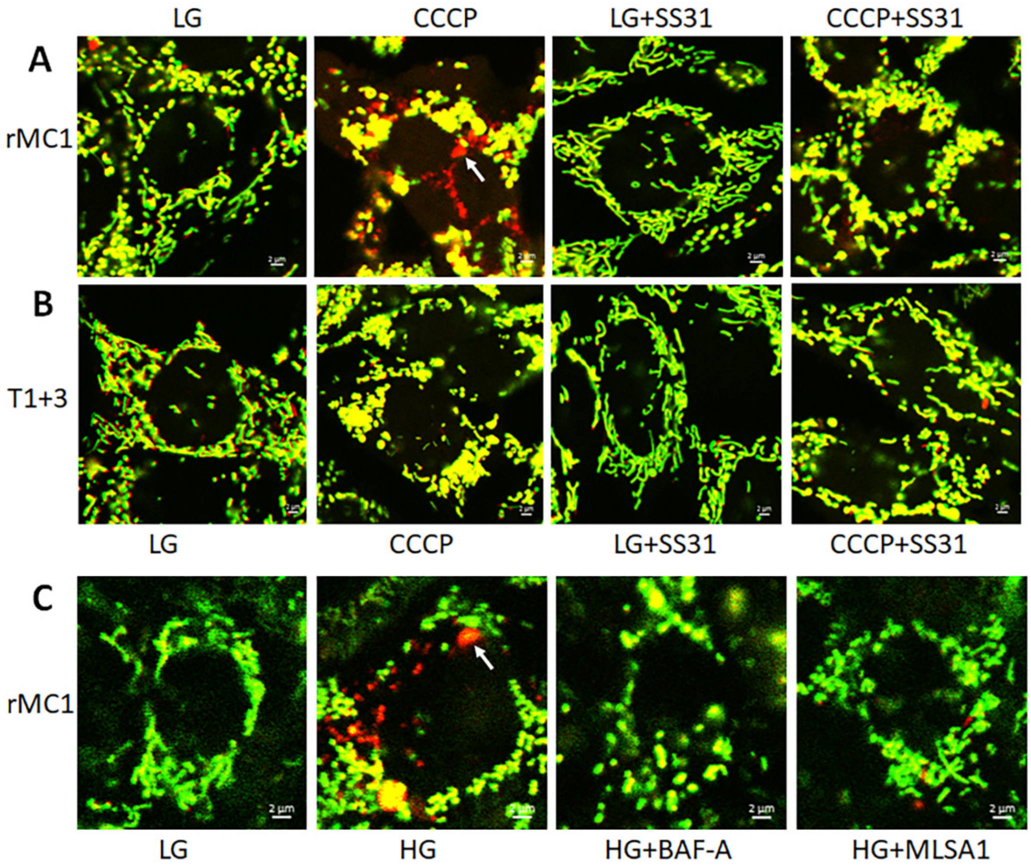

Figure S4. Mitochondria and lysosome targeted drugs prevent mitophagic flux in rMC1. (A) rMC1 cells were transduced with Ad-CMV-mt-Keima (vector builders) for 3 days and treated with carbonyl cyanide m-chlorophenyl hydrazone ionophore (CCCP, 20 μM) for 24 h with or without mitochondrial anti-oxidant SS-31, and compared with that in LG alone (control). CCCP induced mitophagic flux in rMC1 as red mt-Keima is observed (white arrow) than in LG. Treatment with SS-31 (4μM) prevents red mt-Keima formation, suggesting mitochondrial oxidative stress is involved in CCCP-induced mitochondrial damage and mitophagy. (B) Similarly, CCCP effect is also reduced in TXNIP knockout rMC1 cells, further supporting the role of TXNIP in redox stress and mitochondrial dysfunction. (C) Finally, HG-induced mitophagy is also reduced by lysosomal v-ATPase inhibitor, bafilomycin A (BAF-A, 100nM) and MCOLN1 activator ML-SA1 (20 μM), indicating lysosomal activity is critical in the autophagic/mitophagic flux. A representative of n = 3 live-cell confocal imaging is shown, 630× magnification, captured in a Zeiss confocal microscope.

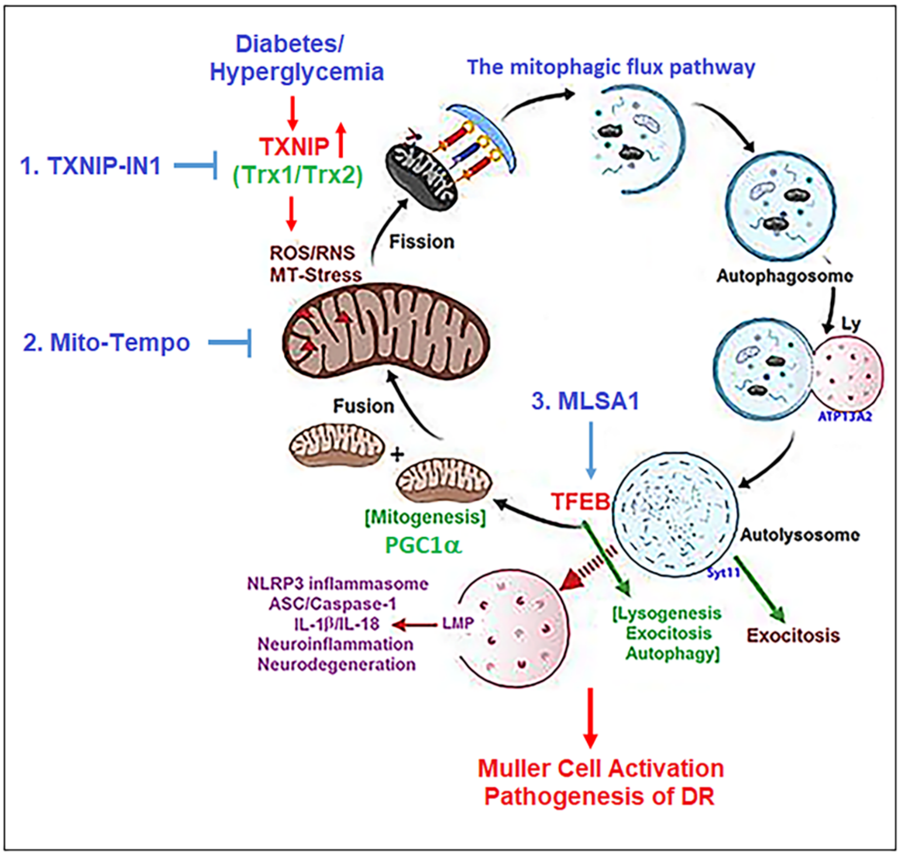

Figure S5. Diagram showing drug targeting the TXNIP-redox and the mitochondrial–lysosomal pathway. TXNIP upregulation in diabetes and under high glucose environment inhibits thioredoxin anti-oxidant system, causing reactive oxygen/nitrogen stress (RO/NS) and mitochondrial dysfunction. Damaged mitochondrial are removed by mitophagy, a specific process of autophagic lysosomal degradation. Unchecked or dysregulation of mitophagy leads to accumulation of damaged mitochondria, which release mtROS and oxidized mtDNA that are inflammatory. Therefore, we propose that multiple steps, targeting the redox stress and mitochondrial–lysosomal quality control cycle will be beneficial in ameliorating DR pathogenesis. Hence, a combination of TXNIP-IN1 (an inhibitor of TXNIP-Trx interaction, 5 μM), Mito-Tempo (a mitochondria-targeted anti-oxidant, 5 μM), and ML-SA1 (lysosomal mucolipin 1 agonist, 20 μM) were used in this study. Figure adapted from ref. 14. (Created with BioRender.com).

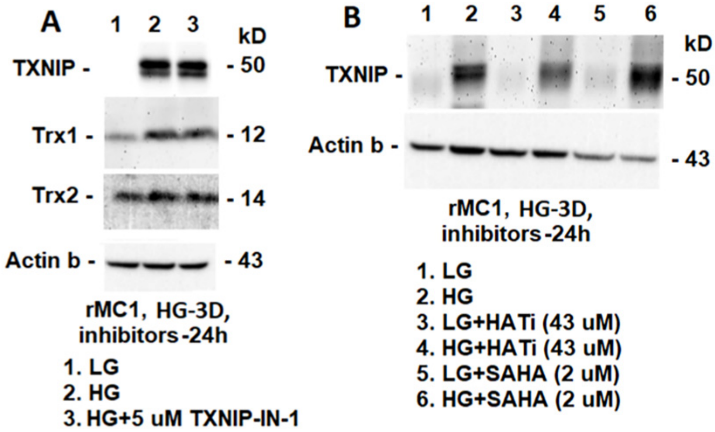

Figure S6. TXNIP-IN1 does not reduce HG-induced TXNIP expression in rMC1. (A) On Western blots, TXNIP expression increases in rMC1 under HG versus LG; however, TXNIP = IN-1 (5 μM) has no effect. Conversely, histone acetyltransferase (HAT) inhibitor (HATi, 43 μM; HAT Inhibitor VIII, cat# 5005110001, Sigma) reduces HG-induced TXNIP levels, while (B) histone deacetylase inhibitor (SAHA, 2 μM; cat# SML0061, Sigma) increases TXNIP expression. Actin β was used as a protein loading control. We have previously demonstrated that TXNIP transcription is regulated by histone acetylation (H3K9Ac, H4K8Ac; ref. 8,12). A representative of n = 2 blots is shown both for (A) and (B).

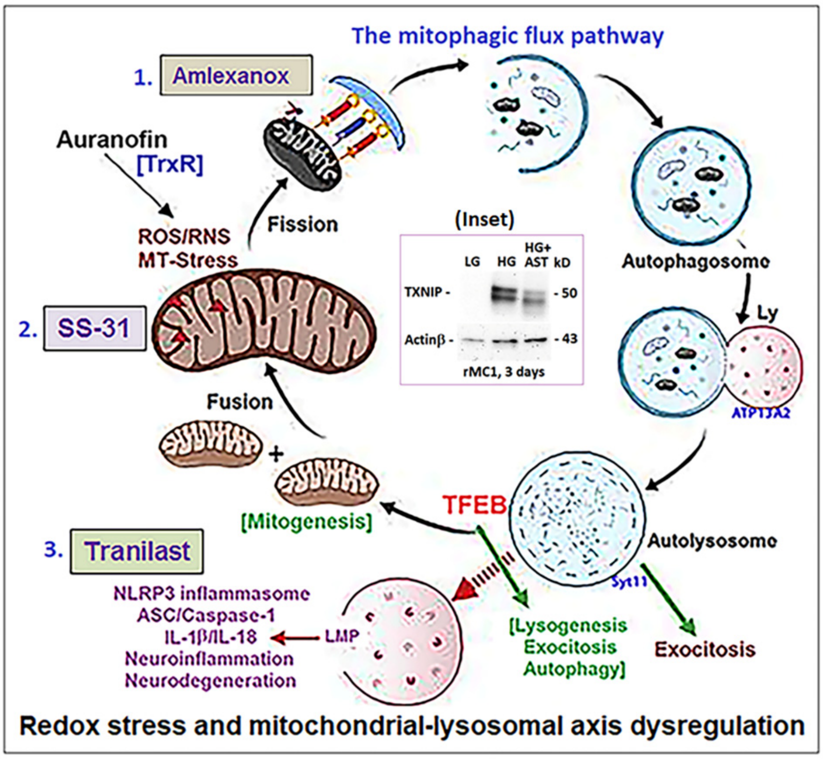

Figure S7. A potential combination drug therapy for redox stress-induced mitochondrial–lysosomal axis dysregulation and inflammasome activation in retinal diseases. Auranofin, which inhibits the redox protein thioredoxin reductase (TrxR) induces redox stress, mitochondrial dysfunction, mitophagy flux, lysosome destabilization, and NOD-like NLRP3 inflammasome activation (14). Therefore, drugs that regulate the mitochondrial cycle may have protective effects in maintaining the mitophagy–lysosomal pathway under cellular stress. We tested in the present study a combination of chemical inhibitors targeting this pathway, which includes amlexanox (an inhibitor of TBK1 kinase and mitophagy adaptors optineurin/p62 phosphorylation, 4 μM), SS-31 (a mitochondria-targeted anti-oxidant, 4 μM), and tranilast (an inhibitor of TXNIP and NLRP3 inflammasome, 56.5 μM; ref. 57,58). Interestingly, these three drug combinations (AST) also reduces HG-induced TXNIP-upregulation in rMC1 (inset, representative of n = 2). Figure adapted from ref. 14. (Created with BioRender.com).

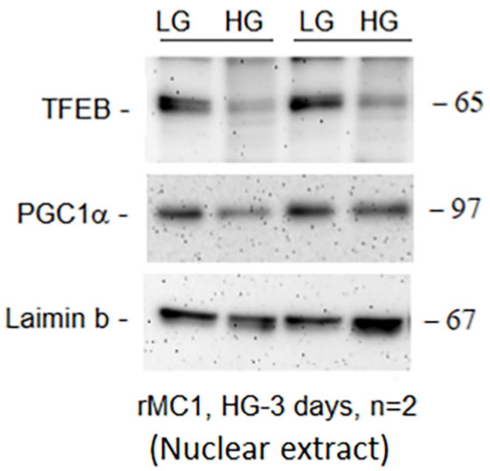

Figure S8. HG increases TXNIP expression and reduces transcription factors TFEB and PGC1α in rMC1. On Western blots, the nuclear levels of transcription factor TFEB and PGC1α are reduced in rMC1. Lamin B1 (Cat# 12586, cell signaling) is used as a nuclear protein loading control. Thirty μg of proteins was loaded in each gel (n = 2).
